# Vascular Injury After Scoliosis Correction in Ehlers-Danlos Syndrome: Proceed With Caution

**DOI:** 10.5435/JAAOSGlobal-D-23-00061

**Published:** 2023-08-15

**Authors:** Gautham Prabhakar, Rishi K. Gonuguntla, David Momtaz, Christopher Chaput, Grant D. Hogue

**Affiliations:** From the Department of Orthopaedics, UT Health San Antonio (Dr. Prabhakar, Gonuguntla, Momtaz, Dr. Chaput), San Antonio, TX (Dr. Prabhakar, Dr. Gonuguntla, Dr. Momtaz, and Dr. Chaput); and the Department of Orthopaedics, Boston Children's Hospital/Harvard Medical School, Boston, MA (Dr. Hogue).

## Abstract

Ehlers-Danlos syndrome (EDS) is a rare inherited connective tissue disorder characterized by collagen synthesis disruption, resulting in joint hyperlaxity, skin and vascular fragility, and bleeding diathesis. Patients with EDS are susceptible to spinal deformities, with scoliosis accounting for up to 23.4% of musculoskeletal abnormalities. Conservative management is often trialed initially; however, severe scoliosis can lead to significant sagittal imbalance and cardiopulmonary compromise. Surgical intervention for scoliosis correction in patients with EDS presents unique challenges because of tissue fragility and an increased risk of vascular and wound complications. This case report discusses a 20-year-old man with type II EDS and scoliosis, who experienced retroperitoneal compartment syndrome, significant left lower extremity weakness, and loss of sensation after scoliosis correction surgery. The report also provides an overview of the existing literature on scoliosis surgery outcomes in patients with EDS, highlighting the need for heightened vigilance and cautious surgical approaches.

Ehlers-Danlos syndrome (EDS) is an inherited connective tissue disorder caused by collagen synthesis disruption leading to skin and vascular fragility, bleeding diathesis, and joint hyperlaxity.^1^ Owing to these collagen-related defects resulting in hypermobility, patients with EDS are especially susceptible to spinal deformities, and scoliosis accounts for up to 23.4% of musculoskeletal conditions in this vulnerable cohort.^2,3^ Often, conservative management is initially trialed; however, as instability ensues, the scoliosis becomes more severe, leading to significant sagittal imbalance and cardiopulmonary compromise.^4–6^

Patients with EDS are at increased risk of vascular and wound complications owing to their tissue fragility.^7,8^ This brings notable challenges to the spine surgeon, and the literature investigating scoliosis correction surgery in pediatric patients with EDS is sparse. Although some studies have suggested safety measures such as using desmopressin to control bleeding, hypotensive anesthesia, avoiding blunt dissection, and limiting diskectomy, the risks of this surgery in patients with EDS should be constantly at the forefront and managed meticulously.^9–11^

As this pathology is not commonly encountered by the spine surgeon, the authors think it is imperative that the clinician be cognizant of the significant vascular complications that can occur during scoliosis correction. A case of scoliosis correction in a patient with type II EDS that was complicated by retroperitoneal compartment syndrome secondary to left internal iliac artery bleeding and subsequent left lower extremity (LLE) muscle weakness with loss of sensation is detailed in this report.

## Case Report

**Figure 1:**
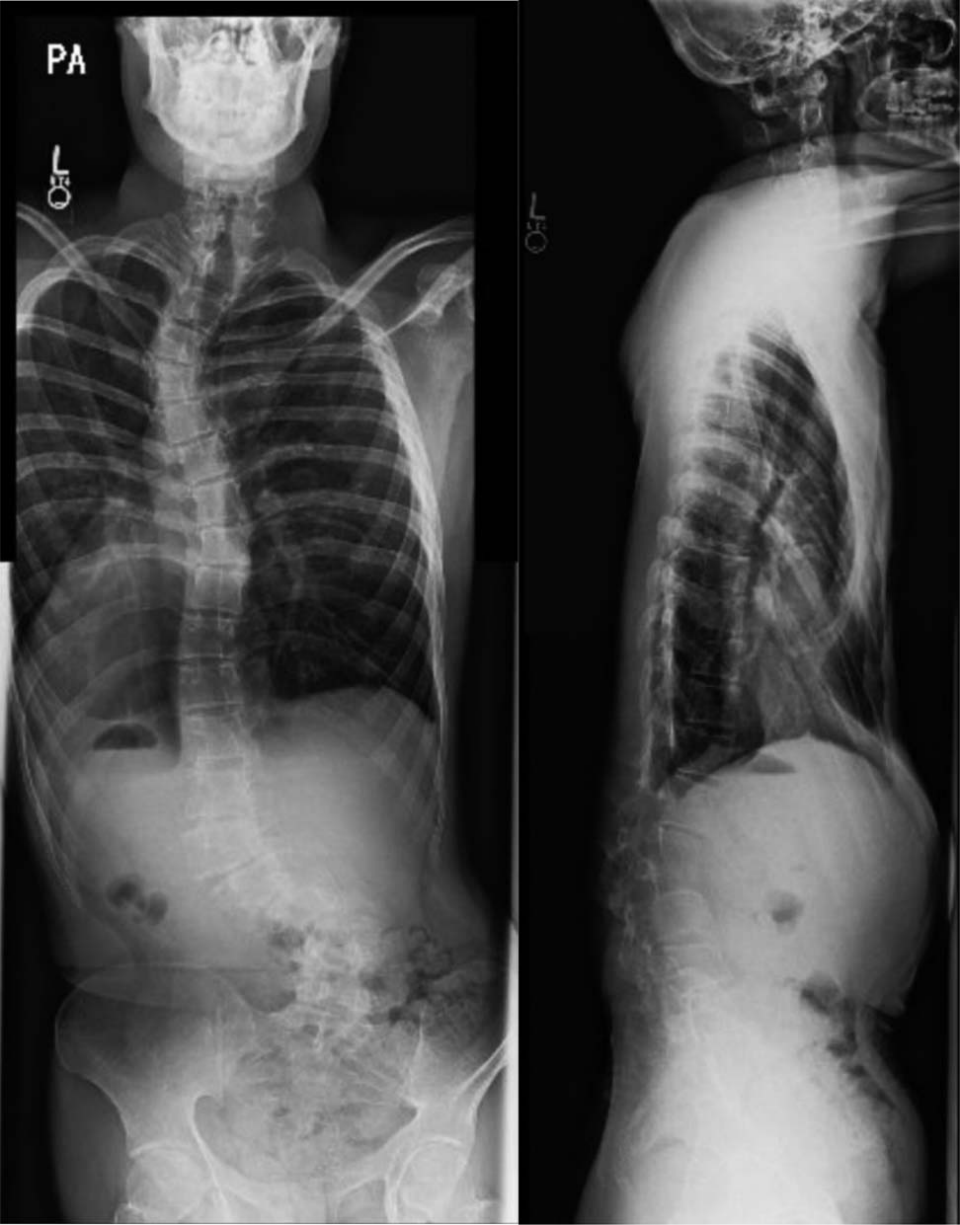
Posteroanterior and lateral plain radiographs of the initial presentation demonstrating lumbar kyphosis and leftward listhesis of L2-3 and L3-4.

The patient is a 20-year-old man with a history of type II EDS and scoliosis that was previously managed for 3 years with a brace, presenting to our institution for severe, worsening scoliosis deformity with new-onset lower back pain with prolonged sitting and standing activities. On presentation, plain radiographs revealed notable curves in the spine: a left upper thoracic curve of 45°, a right thoracic curve of 30°, a left lumbar curve of approximately 45°, and significant lumbar kyphosis with a trunk shift to the left. In addition, there was significant leftward listhesis of L2-3 and L3-4 (Figure 1). Multiplanar, multisequence magnetic resonance imaging (MRI) without contrast of the cervical, thoracic, and lumbar spine showed no significant intrathecal abnormality. A spinal fusion to correct the progressive deformity was indicated.

The patient was positioned prone on a Jackson table after general anesthesia was induced. A midline incision from T10 to S2 was made while preserving the interspinous ligament cranially, followed by sharp dissection with electrocautery to reveal the posterior elements. Facetectomies were performed from T10 to S1. Pedicle screws were placed at levels amenable to this modality, whereas sublaminar bands were used at levels where pedicle anatomy was inadequate for screw passage. A combination of fluoroscopy and navigation was used for screw placement. All screws were interrogated to ensure no breach, and there were no neuromonitoring abnormalities. Rods were then placed with standard corrective maneuvers. There were no intraoperative complications immediately encountered, and the patient was admitted to the pediatric intensive care unit. Postoperatively, there were no motor or sensory deficits, and his examination was consistent with his preoperative baseline. Of note, the patient had a hemoglobin level of 10.8 and a hematocrit level of 32 on completion of the procedure.

Overnight, the patient became tachycardic and hypotensive with reports of loss of sensation about the LLE. He was repositioned and quickly became diaphoretic, confused, and pale. Blood gas revealed acute metabolic acidosis with a hemoglobin level of 6.8. The patient received one unit of packed red blood cells over 1 hour over which he had immediate resolution of symptoms. His vitals stabilized with complete return of sensation throughout the LLE. Approximately 3 hours later, the patient again reported confusion, dyspnea, diaphoresis, and numbness throughout the LLE. The patient exhibited progressive abdominal distention with clinical symptoms of acute hypovolemic shock. A massive transfusion protocol was initiated, and the patient was taken to the operating room for exploratory laparotomy. The laparotomy showed extensive hematoma with active bleeding from the retroperitoneal space. The abdomen was packed with laparotomy sponges for hemostasis, and the patient was transported to the interventional radiology suite. Arteriogram revealed avulsion of several leading branches of the left internal iliac artery, resulting in significant extravasation. Gel foam embolization of the left internal iliac artery was successful with no residual extravasation. He then returned to the operating room for removal of the laparotomy sponges and irrigation and evacuation of remaining hematoma. A negative pressure wound therapy was used for the wound. Overall, the patient received 26 units of packed red cells. His abdomen was closed 6 days later. Postoperatively, and for the duration of his stay, the patient experienced significant weakness globally in all distributions of the LLE with decreased sensation in the L2-S1 dermatomes. Although extremely rare, it is the authors' belief that this was a result of a retroperitoneal compartment syndrome. Owing to this, the patient required a prolonged hospital stay for approximately 3 weeks. Shortly after discharge, the patient required readmission because of a deep vein thrombosis of the left common femoral, popliteal, and greater saphenous veins, which was treated with therapeutic anticoagulation.

**Figure 2:**
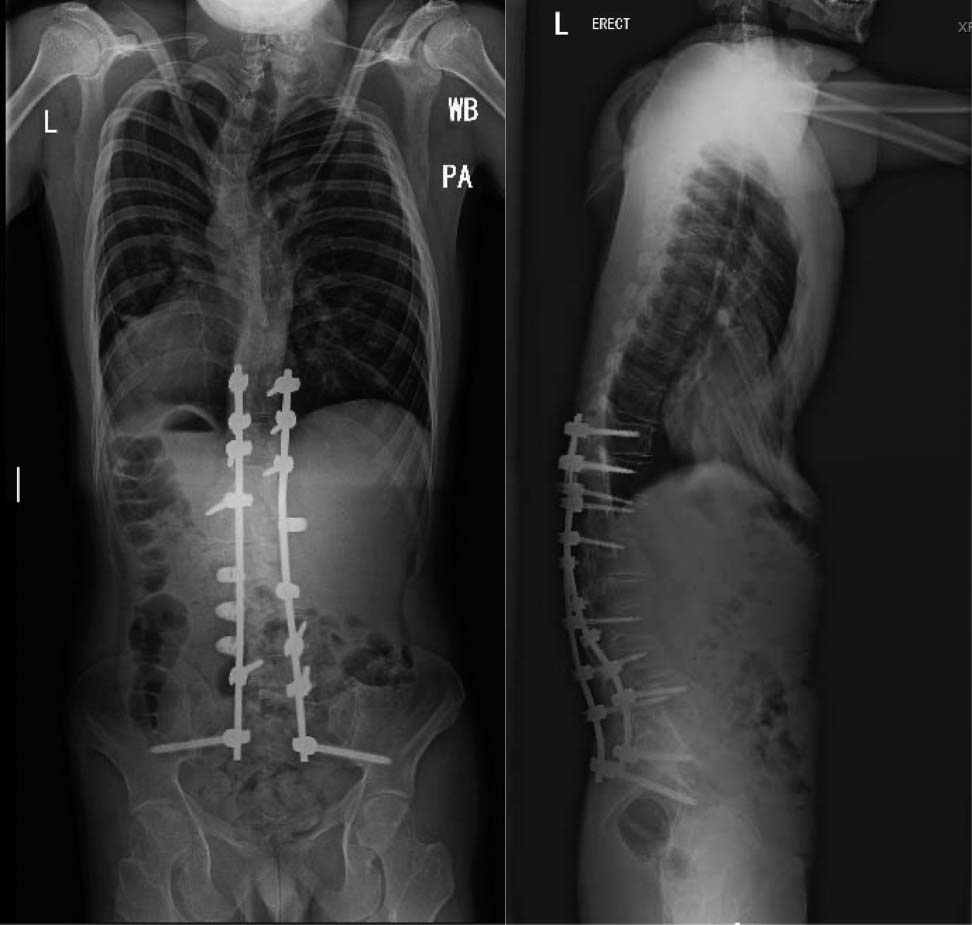
Posteroanterior and lateral plain radiographs demonstrating stable proximal junctional kyphosis and construct at 3-year follow-up.

At the 1-month follow-up visit, the patient reported a 40% return of sensation in the L2-S1 dermatomes. His LLE remained quite weak, with 0/5 tibialis anterior (TA) and extensor hallucis longus (EHL) strength and 2/5 strength in the quadriceps and adductors. At the 6-month mark, the patient had gained more proximal strength but continued with only a flicker of the TA and no motion of the EHL. He was able to ambulate without assistive devices for short distances, which was a dramatic improvement from previous visits where he had been using walker constantly. At 1-year follow-up, the patient continued to have left foot dysesthesias albeit improved from the index procedure. He was ambulating with a Canadian crutch independently but continued with weak TA and EHL function. At the most recent 3-year follow-up, the patient's gait markedly improved with no footdrop, and he no longer required a cane or brace. His visual analog scale (VAS) pain score was zero, and the patient started undergraduate studies. His left quadriceps muscles returned to normal muscle mass, and he had much improved TA function with 4/5 strength (Table 1). The initial neurapraxia of the LLE had essentially resolved with only some diminished sensation in the dorsum of the foot. Radiographically, the patient had a stable proximal junctional kyphosis of 18°, which is currently asymptomatic (Figure 2).

**Table 1:** Motor Scoring at Progressive Follow-up Visits

	1 mo	6 mo	1 yr	2 yr	3 yr
HF	3/5	4/5	4/5	4/5	5/5
KE	3/5	4/5	4/5	4/5	4/5
TA	1/5	1/5	2/5	3/5	4/5
EHL	0/5	0/5	0/5	1/5	2/5
GS	2/5	3/5	3/5	3+/5	4/5

EHL = extensor hallucis longus, GS = gastrocnemius-soleus complex, HF = hip flexion, KE = knee extension, TA = tibialis anterior.

## Discussion

The constellation of tissue and vascular fragility, joint laxity, and muscle hypotonia seen in patients with EDS presents a difficult set of challenges in the setting of spine surgery. The loose ligamentous structures along with muscle hypotonia contribute to rapid scoliosis progression and large degree curves. Although nonsurgical management is usually initially trialed, it is often unsuccessful.^12^

The existing literature examining the relationship and outcomes of EDS and spine surgery is very limited. In 1988, Leatherman and Dickson^13^ warned readers to avoid surgery because the risk of the bleeding and poor soft-tissue healing that occur in these patients can be catastrophic. However, conservative management often fails, and rapid curve progression may lead the surgeon to offer invasive treatment. Although it is established that vascular injury can be a serious complication of spinal surgeries, leading to the development of new neurological symptoms, these are quite rare.^14^ A review of 19,360 cases of pediatric scoliosis corrections by Reames et al^15^ found that 0.5% of patients experienced a nonfatal hematological complication, and 0.01% of patients experienced the development of a deep vein thrombosis. Akesen reported that in their cohort of 85 patients undergoing scoliosis correction, 13 were admitted to the intensive care unit after their surgery, but that only one of the patients cited hematological instability as the reason for admission.^16^ Similarly, Aguirre et al^17^ found that none of their 260 pediatric and adult patients undergoing scoliosis correction experienced a major bleeding event, and that only 1.5% of patients experienced a minor bleeding event. However, the situational circumstances differ when dealing with patients suffering from EDS.

A high risk of neurological and vascular complications exists when correcting scoliosis in patients with EDS.^11^ Several cases of vascular injury after spinal surgery to correct scoliosis in patients with EDS are documented in the literature. Vogel and Lubicky^18^ reported four cases of neurological and vascular complications because of scoliosis surgery in this subset of patients. Importantly, one patient in their cohort experienced avulsion of the segmental arteries during anterior spinal surgery, which required repair of the aorta. Two patients developed paraplegia, and one experienced unilateral foot/ankle weakness with a transient neurogenic bladder. Akpinar et al^11^ described five patients with EDS who failed brace treatment requiring surgical intervention for a mean lumbar and thoracic preoperative curve of 69.7° and 46.4°, respectively. One patient in their cohort experienced a rupture of the common iliac artery and vein during blunt dissection that could have been fatal. Luckily, with grafting of the aorta and iliac artery and ligation of the vein, the patient had no long-term sequelae.^11^ Uehara et al^19^ experienced large amounts of blood loss despite administration of intranasal vasopressin because of tissue frailty in a patient with EDS undergoing spinal surgery for kyphoscoliosis. There are several reports of vascular injury in patients with EDS in the literature. However, this study is the first to document long-term neurological sequelae secondary to complications from spine surgery in a patient with EDS.

By contrast, Pozdnikin and Ryzhakov^20^ reported no significant neurological or vascular injury in their surgical correction of eight EDS patients with kyphoscoliosis. Echoing these results, Jasiewicz et al^5^ had a similar experience, with none of their 11 patients with EDS suffering from vascular or neurological complications. Given the rarity of EDS, there are very few large studies that assess postoperative outcomes after spine surgery in this cohort. Matur et al^21^ conducted a retrospective review of 21,490 patients from the National Surgical Quality Improvement Program (NSQIP) database between 2012 and 2016, and found that there was no notable difference in the rates of bleeding complications after spinal surgery in pediatric patients with and without EDS. Although this is the largest study investigating the differences in rates of vascular complications, the study cohort included only 56 patients with EDS.^21^ In addition, the NSQIP database is limited by a lack of radiographs, a detailed history, and what surgical approach/method was used, as well as whether there were any specific intraoperative complications or other concurrent concerns. Furthermore, outcomes longer than 30 days out are usually not tracked within the NSQIP database, limiting a full picture analysis.

The patient's unusual presentation of severe LLE weakness and sensory deficits after a vascular insult is suspicious for retroperitoneal compartment syndrome. Abdominal/retroperitoneal compartment syndrome is a life-threatening condition caused by elevated intra-abdominal pressure that is treated with explorative laparotomy.^22^ Although rare, this has been a documented complication after scoliosis correction and has been reported in the literature.^23,24^ There are no official guidelines in preventing catastrophic vascular injury in these vulnerable patients. Preoperative angiography is usually of little benefit because many of these patients do not have structural defects or occlusions.^8^ Catching these complications becomes even more difficult when there is no direct indication of injury intraoperatively or immediately postoperatively. Possible risk factors that may influence a physician to be aware of the potential for abdominal compartment syndrome include high-volume fluid resuscitation, organ failure, prone positioning, and hypotension.^23^ In this particular case, it is possible that a high-risk complication may have been mitigated by limiting the degree to which the deformity was corrected in this patient with EDS. One way to do so may be to correct the deformity to the level achieved on preoperative bending radiographs.^9^ Postoperative vascular complications can occur >8 to 12 hours after the procedure, and thus, the surgeon must keep high vigilance when dealing with this unique patient cohort.

## Conclusion

Conservative management for scoliosis in patients with EDS often fails, requiring surgical intervention. In patients with vascular fragility, special attention must be paid to the amount of correction attempted because devastating complications can occur. Keeping these imperative nuances in this unique patient cohort at the forefront, the surgeon must keep high vigilance and proceed with caution.
